# Neuropathobiology of COVID-19: The Role for Glia

**DOI:** 10.3389/fncel.2020.592214

**Published:** 2020-11-11

**Authors:** Marie-Eve Tremblay, Charlotte Madore, Maude Bordeleau, Li Tian, Alexei Verkhratsky

**Affiliations:** ^1^Axe Neurosciences, Centre de Recherche du CHU de Québec, Université Laval, Québec City, QC, Canada; ^2^Neurology and Neurosurgery Department, McGill University, Montréal, QC, Canada; ^3^Department of Molecular Medicine, Université Laval, Québec City, QC, Canada; ^4^Division of Medical Sciences, University of Victoria, Victoria, BC, Canada; ^5^Department of Biochemistry and Molecular Biology, The University of British Columbia, Vancouver, BC, Canada; ^6^Univ. Bordeaux, INRAE, Bordeaux INP, NutriNeuro, UMR 1286, Bordeaux, France; ^7^Department of Physiology, Faculty of Medicine, Institute of Biomedicine and Translational Medicine, University of Tartu, Tartu, Estonia; ^8^Psychiatry Research Centre, Peking University Health Science Center, Beijing Huilongguan Hospital, Beijing, China; ^9^Faculty of Biology, Medicine and Health, The University of Manchester, Manchester, United Kingdom; ^10^Achucarro Center for Neuroscience, IKERBASQUE, Basque Foundation for Science, Bilbao, Spain; ^11^Department of Neurosciences, University of the Basque Country Universidad del País Vasco/Euskal Herriko Unibertsitatea, Leioa, Spain

**Keywords:** COVID-19, SARS-CoV-2, immunity, astrocyte, microglia, ageing, comorbidity, development

## Abstract

SARS-CoV-2, which causes the Coronavirus Disease 2019 (COVID-19) pandemic, has a brain neurotropism through binding to the receptor angiotensin-converting enzyme 2 expressed by neurones and glial cells, including astrocytes and microglia. Systemic infection which accompanies severe cases of COVID-19 also triggers substantial increase in circulating levels of chemokines and interleukins that compromise the blood-brain barrier, enter the brain parenchyma and affect its defensive systems, astrocytes and microglia. Brain areas devoid of a blood-brain barrier such as the circumventricular organs are particularly vulnerable to circulating inflammatory mediators. The performance of astrocytes and microglia, as well as of immune cells required for brain health, is considered critical in defining the neurological damage and neurological outcome of COVID-19. In this review, we discuss the neurotropism of SARS-CoV-2, the implication of neuroinflammation, adaptive and innate immunity, autoimmunity, as well as astrocytic and microglial immune and homeostatic functions in the neurological and psychiatric aspects of COVID-19. The consequences of SARS-CoV-2 infection during ageing, in the presence of systemic comorbidities, and for the exposed pregnant mother and foetus are also covered.

## Introduction

Invasion of the SARS-CoV-2 into the lung is the first, and the most frequent, step in COVID-19. Yet the virus, and the immune responses against it, can spread into the other organs including the central nervous system (CNS) and cause severe neurological outcomes during the disease development and progression, especially for patients with comorbidities and/or elderly people (Huang et al., 2020; Wolfel et al., 2020; Wu and McGoogan, 2020).

The Coronavirus Disease 2019 (COVID-19) pandemic is the second major virus outbreak that engulfs the world threatening mankind and disrupting the canvas of civilisation. Rather ominously, the first one was a Spanish flu caused by influenza A virus subtype H1N1 that started in the summer of 1918; the influenza pandemic added to the global world catastrophe instigated by the Great War and claimed ~50 million people, being thus two times deadlier than the mass killing on the battlefield (Morens and Fauci, 2007). The clinical presentation of Spanish flu included neurological and neuropsychiatric symptoms, both acute and delayed; the H1N1 virus was arguably linked to the still mysterious encephalitis lethargica, which alone claimed 500,000 death in 1920s (Crookshank, 1919; Ravenholt and Foege, 1982). Similarly, the clinical presentation of COVID-19 frequently includes neurological symptoms and neuropathologies such as acute ischemic stroke, meningitis/encephalitis, acute necrotising haemorrhagic encephalopathy, acute Guillain–Barré syndrome (Beyrouti et al., 2020; Dixon et al., 2020; Oxley et al., 2020; Paterson et al., 2020; Pero et al., 2020; Poyiadji et al., 2020; Zhao et al., 2020) as well as psychiatric manifestations such as depression, delirium, and psychosis (Steardo et al., 2020b). The retrospective analysis revealed that up to 20–30% of patients with severe forms of COVID-19 presented signs of disrupted consciousness and altered mental status (Chen et al., 2020; Varatharaj et al., 2020).

In this review, we discuss the neurotropism of SARS-CoV-2, the implication of neuroinflammation, adaptive and innate immunity, autoimmunity, as well as glial cells (mainly astrocytes and microglia) in the neurological and psychiatric aspects of COVID-19. The consequences of SARS-CoV-2 infection during ageing, in presence of comorbidities, and for the exposed pregnant mother and foetus will be specifically covered. We will also highlight promising research avenues to pursue regarding the involvement of glial cells in COVID-19 neuropathobiology.

## The Neurotropism of SARS-COV-2

**Figure 1:**
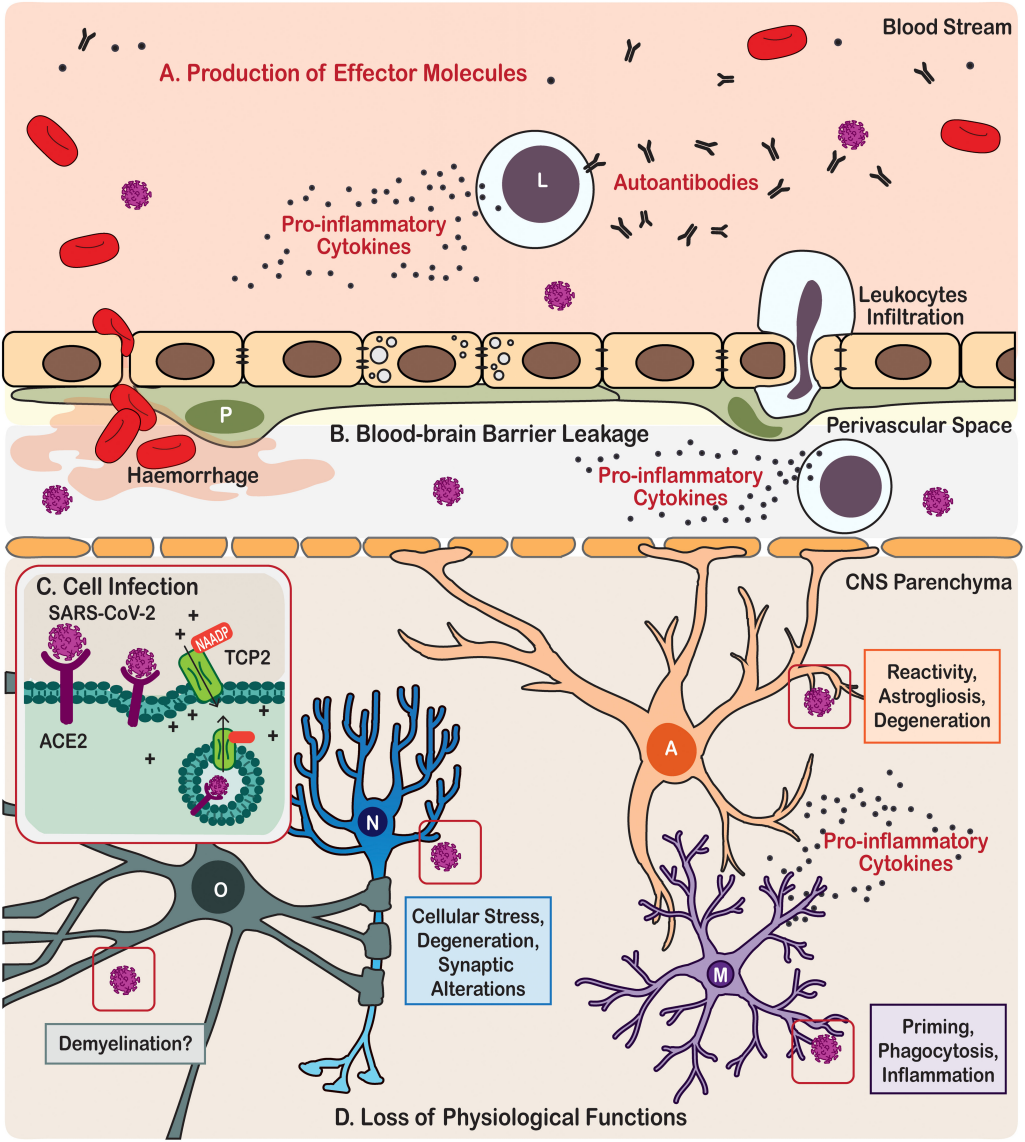
Scheme illustration of the neurotropism, neuroinflammatory processes, and effects on brain cells triggered by COVID-19 in patients. **(A)** Immune cells from the periphery and the central nervous system (CNS) produce effector molecules that include pro-inflammatory cytokines and autoantibodies. **(B)** SARS-CoV-2 infection also causes leakage of the blood-brain barrier leading in some cases to haemorrhage and cerebral infarct, as well as eliciting leukocytes infiltration. **(C)** In the parenchyma, the CNS cells become infected by SARS-CoV via angiotensin-converting enzyme 2 (ACE2) endocytosis mediated by the two-pore channel 2 (TCP2). **(D)** SARS-CoV-2 infection leads to loss of physiological functions of the brain cells, including neurones, astrocytes, microglia, and oligodendrocytes. Cell types are identified in the following manner; A, Astrocyte; L, Leukocyte; M, Microglia; N, Neurone; O, Oligodendrocyte.

How SARS-CoV-2 damages the CNS? First and foremost, the virus can directly infect neural cells (Song et al., 2020). The SARS-CoV-2 belongs to the group 2B of β-coronavirus family, several members of which demonstrate neurotropism (Lau et al., 2004; Xu et al., 2005; Steardo et al., 2020a; Zhou et al., 2020). There are several routes for coronaviruses entry to the CNS. The most studied pathway for infection involves binding to the receptor angiotensin-converting enzyme 2 (ACE2), which together with other components of angiotensin system is expressed in the CNS, mostly by endothelial cells (Zeisel et al., 2015) but also by both neurons and neuroglial cells (Xia and Lazartigues, 2010; Gowrisankar and Clark, 2016; Tekin et al., 2018; Nemoto et al., 2020). Like a key inserted into a lock, ACE2 permits SARS-CoV-2 to invade cells using the spike-like proteins located on their surface. The actual invasion occurs through endocytosis controlled by endosomal proton pump, and by NAADP-sensitive intracellular two-pore channel 2 (TPC2) (Petersen et al., 2020) (Figure 1). The ACE2 is particularly concentrated in the brain stem, among the regions associated with regulation of cardiovascular and respiratory systems. In particular, ACE2 populates the circumventricular organs (CVOs), the subfornical organ, paraventricular nucleus (PVN), nucleus of the tractus solitarius (NTS), and rostral ventrolateral medulla (Xia and Lazartigues, 2010; Gowrisankar and Clark, 2016). All these structures are highly vascularised and they lack the blood-brain-barrier (BBB) (Duvernoy and Risold, 2007), which is formed by the endothelial cells lining brain vessels, together with pericytes, astrocytes, microglia, and extracellular components such as the basement membrane that together form the neurovascular unit (Abbott et al., 2006; Grotta et al., 2008; del Zoppo, 2009; Iadecola, 2017). Absence of the BBB, crucial for the constant monitoring of peripheral health status, makes CVOs more vulnerable to peripheral neurotoxic molecules or invasive agents. Coronaviruses may also infect CNS structures following intranasal administration; possibly with the trans-synaptic route being the main culprit (Netland et al., 2008; Li et al., 2016). Trans-nasal and trans-synaptic invasion in particular seem to deliver the viral particles into the olfactory bulb and then into the brain stem, possibly compromising the respiratory centres (Li et al., 2016). Post-mortem magnetic resonance imaging (MRI) analysis in COVID-19 patients demonstrated an asymmetry in olfactory bulb and aberrant brain parenchyma in 21% of samples thus further corroborating the relevance of trans-nasal CNS infection (Coolen et al., 2020).

Besides direct invasion of neural cells, SARS-CoV-2 virus affects the neurovascular unit and CNS through systemic inflammation. The major feature of the systemic infection in COVID-19 is the massive increase of pro-inflammatory factors in the circulating blood, often described as a “cytokine storm” (Coperchini et al., 2020) (Figure 1). Although the BBB segregates the CNS from the systemic circulation, this segregation is not absolute. The brain and spinal cord constantly communicate with the body's immune system; systemic inflammation, when developed, inevitably influences the CNS (Schwartz and Deczkowska, 2016). Both innate and adaptive immune responses can affect the CNS, through their cellular elements and circulating pro-inflammatory factors. For example, effectors T- and B-lymphocytes enter the CNS (even when the BBB stays intact) aiming at invasive pathogens (Hickey et al., 1991; Carson et al., 2006). Leucocytes enter the CNS through the choroid plexus and disseminate through the perivascular space (Wilson et al., 2010). Systemic infection which accompanies severe cases of COVID-19 was shown to proceed with substantial increase in circulating levels of chemokines and interleukins, which compromise the BBB and enter the CNS where they encounter the defensive systems represented by astrocytes and microglia. Performance of these immune and glial cells is critical in defining the neurological damage and neurological outcome of the COVID-19.

## Astrocytes and Microglia: The Gatekeepers and Defenders of the CNS

**Figure 2:**
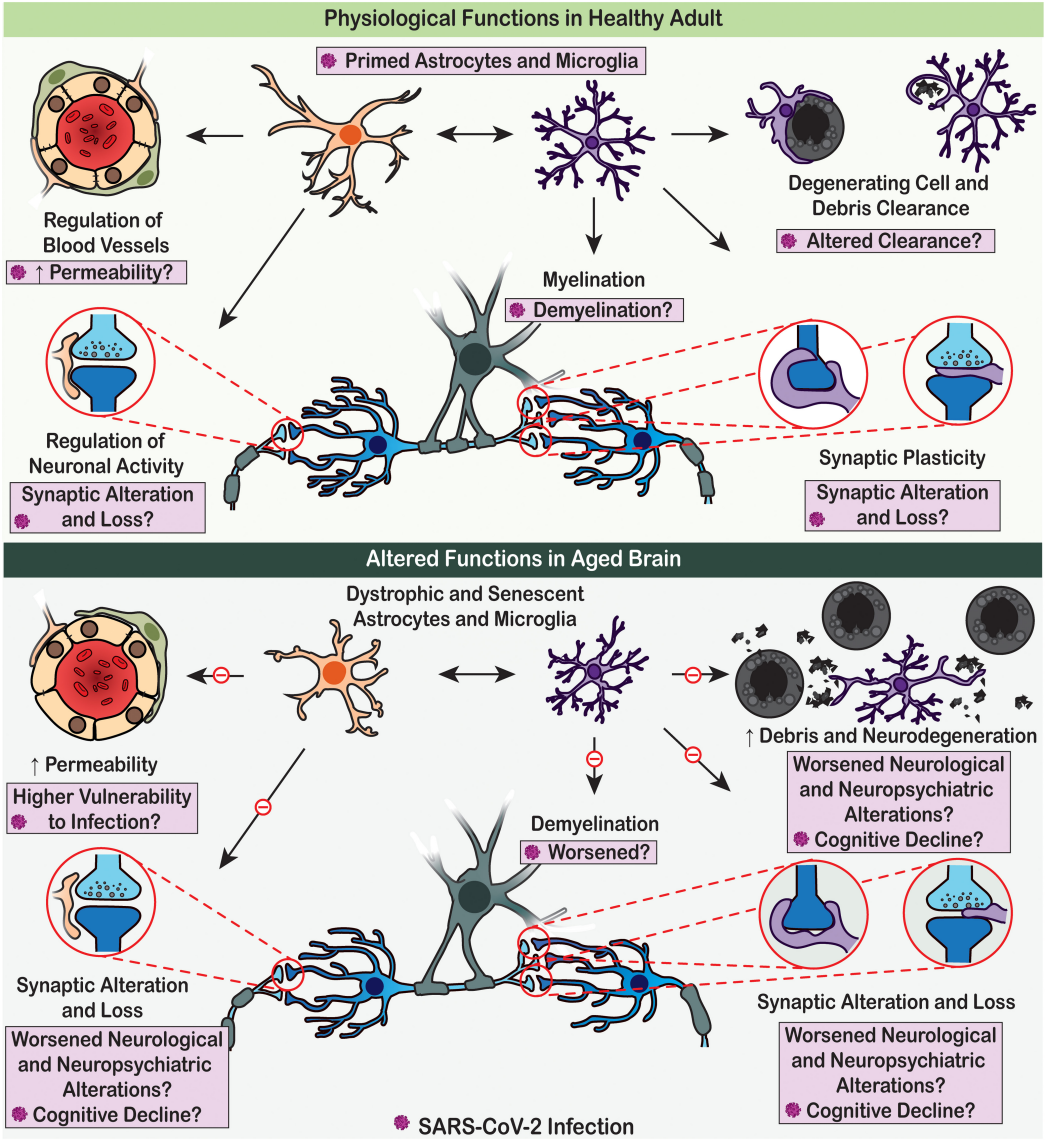
Physiological functions of astrocytes and microglia are altered with age, which may render the brain vulnerable to SARS-CoV-2 detrimental effects. (Upper panel) Under adult homoeostatic conditions, astrocytes notably contribute to the regulation of neuronal activity and neurovascular function (e.g., blood flow and permeability), while microglia participate to myelination, synaptic plasticity, and clearance processes. Upon SARS-CoV-2 infection, these physiological roles are altered, potentially contributing to the COVID-19 associated cognitive and neuropsychiatric symptoms. (Lower panel) In aged brain, dystrophic astrocytes and microglia decrease their efficient contribution to brain homeostasis due to impaired physiological functions. These dysfunctions of glial cells promote neurodegenerative processes, potentially leading to worsened cognitive decline upon SARS-CoV-2 infection.

Astrocytes and microglia are highly heterogeneous cell populations. They are, respectively, neural cells of ectodermic origin and innate immune cells of mesodermal origin, which together represent key elements of the CNS homoeostatic system (Figure 2). Astrocytes and microglia sustain homeostasis ranging from the molecular to the whole organic levels, including via transport of ions, uptake of neurotransmitters, scavenging of reactive oxygen species (molecular level), regulation of neurogenesis, synapse formation, maintenance and elimination (cellular and network levels), regulation of blood flow, glycogen synthesis and storage (metabolic level), as well as control of the BBB and glymphatic clearance (whole brain level) (Hanisch and Kettenmann, 2007; Tay et al., 2017b, 2019; Verkhratsky et al., 2015b, 2016, 2020; Verkhratsky and Nedergaard, 2018; Mestre et al., 2020; Mondo et al., 2020). Astrocytes further contribute to the regulation of energy balance and food intake, blood pH and Na^+^ concentration, as well as of sleep homeostat (Verkhratsky and Nedergaard, 2018).

An important homeostatic task of astroglia is the formation of barriers protecting the brain from the peripheral influences; in essence astrocytes form the nervous tissue that constitutes the BBB. This parenchymal wall is represented by the *glia limitans externa* (bordering the pia matter) and *glia limitans perivascularis* (plastering the intra-parenchymal blood vessels), which are formed by astroglial endfeet (Mathiisen et al., 2010; Quintana, 2017). Astroglial barriers, together with other elements of the BBB, are critical in fencing the brain against both toxic and infectious invasions. Furthermore, astrocytes regulate the permeability of BBB through influencing expression of tight junctions in endothelial cells layer (Kriauciunaite et al., 2020). Serum levels of the astrocytic calcium binding protein S100b, associated with an increased BBB permeability, significantly correlate with COVID-19 disease severity, inflammation markers (ferritin, C-Reactive Protein, procalcitonin), and organ damage markers (alanine aminotransferase, creatinine) (Aceti et al., 2020). S100b is a calcium binding protein, strongly expressed in astrocytic endfeet lining the BBB; genetic deletion of S100b is associated with age-dependent increase in BBB leakage (Wu et al., 2016). Another astrocytic protein that could be affected in the course of SARS-CoV-2 infection is aquaporin-4, also expressed by astrocytic endfeet lining the BBB, and critically involved in the exchange of fluids between the CNS and the vasculature (Nagelhus and Ottersen, 2013).

Juxtavascular microglia contribute to the *glia limitans* with their ramified processes ensheathing the basement membrane, especially in areas devoid of astrocytic endfeet (Bisht et al., 2016; Joost et al., 2019). While microglia are associated with the regulation of vascular remodelling, blood flow and BBB maintenance, the molecular mechanisms underlying these emerging functions are largely undetermined (Zhao et al., 2018; Halder and Milner, 2019; Haruwaka et al., 2019). Among the microglial subtypes that could be involved, “dark microglia,” which are defined ultrastructurally by their cytoplasmic and nucleoplasmic condensation, giving them a dark appearance, were shown to tightly interact with blood vessels. These cells are rare in healthy young adults, but highly prevalent upon maternal immune activation [MIA; notably induced with the viral mimic polyinosinic acid-polycytidylic acid (poly I:C)], chronic stress, ageing, and in various disease states. While dark microglia's consequences on the BBB are still elusive, they make extensive phagocytic interactions with synapses, suggesting their implication in pathological remodelling of neuronal circuits (Bisht et al., 2016; St.-Pierre et al., 2020).

Studies performed in the last decade demonstrated numerous physiological functions of microglia during brain maturation, activity and plasticity throughout life (Tay et al., 2017b, 2019). In critical developmental periods, microglia participate to the brain maturation by regulating glio- and neuronogenesis, synapse formation, maturation and function, by clearing the brain from extracellular debris and by refining its synaptic connections, notably through their phagocytic activity and release of trophic factors, as well as pro- and anti-inflammatory cytokines (Kettenmann et al., 2013; Tay et al., 2017b). Once neuronal circuits are formed, mature microglia play important roles in neuronal circuit remodelling, learning, memory and the adaptation of the brain and behaviour to lifestyle factors including the diet, physical activity, sleep patterns, and other external influences (Garofalo et al., 2020; Madore et al., 2020). More recently, microglia were also found to contribute to developmental myelination and remyelination (for instance in multiple sclerosis) through the release of trophic factors regulating oligodendrocyte progenitor's formation and maturation, as well as removal of myelin debris required for remyelination (Domingues et al., 2016; Hughes and Appel, 2020; Traiffort et al., 2020).

While the consequences of SARS-CoV-2 on cells of the oligodendrocytic lineage responsible for myelination remain unclear, COVID-19 resulted in several cases of demyelinating Guillain-Barré syndrome (Arnaud et al., 2020; Padroni et al., 2020; Sedaghat and Karimi, 2020; Tiet and AlShaikh, 2020; Zhao et al., 2020) and Miller-Fisher syndrome (Gutierrez-Ortiz et al., 2020). Similarly, other recent viral outbreaks were associated with increased cases of Guillain-Barré syndrome and other demyelinating conditions (Beghi et al., 2020). Studies using other coronaviruses, such as herpes virus, revealed that cells of the oligodendrocytic lineage can be infected, yet they survived and contributed to sustaining neuroinflammation (Pan et al., 2020). These effects could be exerted in concert with microglia and astrocytes, as well as regulated by the exposure to peripheral stimuli and other external influences. Astrocytes regulate the activity and reactivity of microglia, and *vice versa*, microglia can regulate the activity and reactivity of astrocytes, also with consequences on cells of the oligodendrocytic lineage. Glial cells are thus orchestrating together the brain health and its response to systemic and local pathology (Pascual et al., 2012; Rothhammer et al., 2018; Jay et al., 2019; Vainchtein and Molofsky, 2020).

## Astrocytes and COVID-19

In systemic infection such as evoked by SARS-CoV-2, the BBB can undergo disruptive or non-disruptive remodelling, so classified based on morphological alterations. The non-disruptive BBB develops through molecular and cellular mechanisms that compromise permeability of the barrier (Varatharaj and Galea, 2017). Disruptive BBB pathology proceeds with anatomical changes, such as loss of tight junctions integrity, increased vesicular transport, re-appearance of fenestrae, degradation of glycocalyx, apoptotic death of endothelial cells, breakdown of glia limitans and astrocytic damage (Minami et al., 1998; Candelario-Jalil et al., 2007; Varatharaj and Galea, 2017). The disruption of the BBB, which is exacerbated by stress, sleep deprivation, metabolic syndrome, vascular comorbidities, as well as ageing, for instance (Małkiewicz et al., 2019), allows CNS entrance of pathological agents, including viral particles among other damage-associated molecular patterns (DAMPs). Infiltration of DAMPs triggers astroglial reactivity, disruption of endfeet and astroglial death thus creating a vicious circle further damaging the BBB (Biesmans et al., 2013; Cardoso et al., 2015). Similarly, microglia respond to BBB disruption and DAMPs presence by increasing their phagocytic capacity and release of pro-inflammatory mediators, together associated with their reactivity (Fleshner and Crane, 2017). The type of BBB pathology depends on the severity of the systemic inflammation and, in case of severe COVID-19 pathology, disruptive compromise of BBB is highly likely.

Apart from keeping the barrier tight, astrocytes contribute to the brain defence by mounting reactive astrogliosis, which is an evolutionary conserved programme of astrocytic remodelling in response to pathological lesions. Activation of astrogliotic programme instigates changes in gene expression, in astrocytic biochemistry, morphology and physiology with an ultimate goal of protecting the CNS against pathological lesion (Sofroniew, 2014, 2015; Pekny et al., 2016; Verkhratsky et al., 2017). Astrogliotic programme is flexible and is disease-specific; numerous investigations demonstrating remarkable diversity of reactive astrocytic phenotypes, incompatible with the much popularised A1/A2 dichotomy (Grubman et al., 2019; Henrik Heiland et al., 2019; Al-Dalahmah et al., 2020; Wheeler et al., 2020). Similarly, the M1/M2 activated microglia nomenclature has been rejected (Ransohoff, 2016). Reactive astrocytes are considered to be essential elements of neuroprotection in infectious diseases: ablation of astrogliosis was shown to exacerbate the brain infection. For example, suppression of astrogliosis in glial fibrillary acidic protein (GFAP) knockout mice substantially facilitates the penetration of *Staphylococcus aureus* or *Toxoplasma gondii* into the brain, which results in vasculitis, purulent ventriculitis and severe brain oedema (Stenzel et al., 2004).

Post-mortem MRI examinations of the brains of patients who died from COVID-19 revealed multiple subcortical haemorrhages (Coolen et al., 2020), which most likely instigated glial reactivity. There are indications for reactive astrogliosis in COVID-19. For example, plasma concentration of the astrogliosis marker GFAP in patients with moderate to severe COVID-19 is significantly increased (Kanberg et al., 2020). The generalised increase in GFAP expression was found in the white matter of a COVID-19 victim with disseminated encephalomyelitis (Reichard et al., 2020). In experimental mouse models of infection with coronavirus, MHV-59A resulted in encephalitis with prominent astrogliosis and induction of astroglial synthesis of cytokines; moreover, neurovirulence correlated with the cytokine response (Li et al., 2004). At the same time, viral infections may damage astrocytes: for example, a severe astrodegeneration in the form of clasmatodendrosis, an astrocytic morphological change characterised by fragmentation of the distal processes and swollen cell bodies, has been identified in the brains of influenza-associated encephalopathy victims (Tachibana et al., 2019). Overall, it is important to analyse how different subtypes of astrocytes and their diverse responses contribute over time to the neurological and psychiatric effects of COVID-19. Determining in animal models the cellular and molecular mechanisms involved in disruptive BBB pathology, astrogliosis promoting neuroprotection upon brain infection, as well as cytokine response and degeneration, in concert with microglial functions, is critical at this stage of investigation.

## Microglia and COVID-19

Microglia, the CNS-resident innate immune cells, are considered to be in a chronic immunologically active alert state at steady-state within the CVOs presenting low BBB protection and high vulnerability to SARS-CoV-2 infection. These microglia have an amoeboid instead of a surveilling (derived from to “surveil,” which means to keep a place or a person under surveillance) morphology (Davalos et al., 2005; Nimmerjahn et al., 2005) and express markers that suggest they are ready to cope with invading agents using phagocytosis and the release of various mediators such as pro-inflammatory cytokines (Takagi et al., 2019). A “primed” or pre-activated microglial state, instigated by previous encounter of inflammatory stimuli, might also lead the CVOs microglia to display an exaggerated response to subsequent challenges, of similar (viral infection with SARS-CoV-2) or different (e.g., psychological stress, gut dysbiosis, metabolic disorders, obesity, ageing, co-morbidities, and other environmental risk factors for disease) nature. Microglial surveillance of the parenchyma, but also contribution to the regulation of neuronal activity, myelination, as well as synaptic plasticity, and cognition, among others essential physiological roles (Salter and Beggs, 2014; Tay et al., 2019), could be reduced or compromised as a result of their resource-consuming immune involvement (Figure 2). Additionally, considering that amoeboid microglia are generally more mobile than surveilling ones (Savage et al., 2019), the migration of infected microglia from the CVOs to other brain regions could significantly contribute to spreading neuroinflammation.

Across the brain, the microglial population comprises different subsets with unique intrinsic properties, performing somehow-specialised physiological functions (Stratoulias et al., 2019; Tan et al., 2020), that can adopt different phenotypes upon exposure to systemic challenges, and could play either complementary or perhaps completely opposing roles upon SARS-CoV-2 infection. Determining how the different microglial subsets, located within the CVOs and other affected brain regions, respond to the infection is important, to identify the neuroinflammatory and/or compromised homeostatic functions to be normalised or restimulated. Studies should assess a panel of microglial phenotypic changes including cellular migration, process motility, phagocytosis, functional interactions with neurones, glial cells and components of the neurovascular unit but also epigenetic, transcriptomic, proteomic and metabolomic modifications, as well as release of mediators (e.g., trophic factors, pro- and anti-inflammatory cytokines). Considering that the microglial phenotypic properties are highly plastic and vary substantially in the course of disease progression, these changes in microglia should be investigated over time, ideally within longitudinal experimental studies, combining animal models and human investigations. This knowledge is crucial for the future design of targeted therapeutic interventions promoting beneficial microglial functions in COVID-19 patients.

As the first responders of the CNS to trauma, injury and disease (Bilbo and Stevens, 2017), microglia are highly sensitive to peripheral metabolism and comorbidities, as well as external influences from the living environment such as the diet, physical activity, and exposure to pollution across life (Tay et al., 2017b; Madore et al., 2020). Depending on the parenchymal history, the cooperation from other brain cells, other components of the neurovascular unit and infiltrating peripheral cells, as well as messages received from the periphery, microglia could play different roles in determining the outcomes of COVID-19 on brain pathology. Even in patients having recovered from SARS-CoV-2 infection, microglia could remain primed via an innate immune memory transformation program mediated by epigenetic changes (Cheray and Joseph, 2018). With ageing, microglia could further become dystrophic or senescent (Streit, 2006; Streit et al., 2009; Spittau, 2017), unable then to perform efficiently their critical surveillance, immune and physiological roles. When and if CVOs microglia become dystrophic, the gate would be open, sentinels would fail and COVID-19 would rage.

Microglial cells affected by stress, gut dysbiosis, metabolic disorders and obesity, physiological and cognitive ageing, and other environmental risk factors, could render individuals more at risk of developing neurological and psychiatric complications from COVID-19, even after complete clinical recovery from the infection. Various challenges from the external environment to which microglia respond, psychological or physiological, are associated with epigenetic, metabolic, proteomic, transcriptomic, densitometric, morphological/ultrastructural changes of these cells that can result in compromised molecular and cellular functions, normally required for brain health, including synaptic function and plasticity, myelination, and BBB maintenance (Tay et al., 2017b; Madore et al., 2020). Dysfunctional or aberrant microglial functions could severely impair cognitive functions, including judgment, decision making, learning and memory (Tay et al., 2017a), and hence might have critical outcomes on the short-, medium- or long-term neurological and psychiatric consequences of SARS-CoV-2 infection that have been reported.

Environmental challenges notably inducing stress are associated with an increased risk of developing major depression, anxiety disorders and schizophrenia, obsessive-compulsive disorder, and several other neurological and neuropsychiatric conditions during life (Schneiderman et al., 2005; Knuesel et al., 2014; Furman et al., 2019). Psychological stress also significantly accelerates the progression of these conditions and exacerbates their symptoms. With ageing, the exposure to stressful events or systemic disorders can also trigger, exacerbate or accelerate cognitive decline, Alzheimer's disease and other forms of dementia. Similarly, viral infections (e.g., with flu and pneumonia) can render individuals more prone to developing depression, psychosis, delirium, Parkinson's disease and other forms of dementia, among other conditions, and even more so with a chronic stress life history (Tate et al., 2014; Bisht et al., 2018). Providing insights into the mechanisms by which microglia exposed to SARS-CoV-2, notably in combination with psychological stress, anxiety and fear resulting from the pandemic situation (e.g., social isolation, disruption of daily activities, work from home, home schooling, etc.), contribute to the neurological and psychiatric aspects of COVID-19 is thus of utmost importance.

In light of these findings highlighting the roles of microglia and their modification by infection and important other environmental risk factors, it will be important to characterise over time the microglial phenotypic transformation in clinically-defined cohorts of COVID-19-affected individuals, while controlling for inter-individual variability. In parallel, using preclinical animal models of SARS-CoV-2 infection, intervention strategies allowing the reprogramming of microglia phenotype in order to promote beneficial surveillance, immune and physiological functions, should be designed and ultimately tested in COVID-19-affected individuals.

## Autoimmunity and Glial Involvement in COVID-19

It is widely recognised that the SARS-CoV-2 virus induces heterogeneous reactions of the immune system, with some patients having none or mild immune responses while others developing cytokine storm and collateral systemic multiorgan damage, which often includes the brain (Chen et al., 2020; Chen and Li, 2020; Huang et al., 2020; Mao et al., 2020). Massive production of cytokines, a characteristic of severe COVID-19, leads to an indiscriminate immune attack on all cells of the human body. Lymphopenia, being another manifestation of a cytokine storm, occurs too, as in critically ill and non-surviving patients the lymphocyte counts were persistently low; conversely in survivors, lymphocyte counts gradually increased over the course of recovery (Huang et al., 2020; Wolfel et al., 2020; Wu and McGoogan, 2020). Sustained severe viral infection can result in both immune deficiencies, due to the burn-out of innate and adaptive immunities, and autoimmunity, due to the loss of self-tolerance by the immune system.

Causes of neurological and/or neuropsychiatric symptoms in a sub-population of COVID-19 patients can be multiple, due to both systemic and intracranial immune responses to SARS-CoV-2, as well as to hypoxia and possibly intensive treatments (Chen et al., 2020; Huang et al., 2020; Mao et al., 2020). As a far-reaching arm for the immune-brain crosstalk, it has been long known that cytokines induced by pathogenic bacteria or viruses cause sickness behaviours that resemble depressive symptoms in animals (Hoogland et al., 2015), and offspring born by MIA pregnant mothers are susceptible for neurodevelopmental, neurological and psychiatric disorders (Bergdolt and Dunaevsky, 2019), as discussed in the next section.

Meanwhile, autoimmunity may be another underlying mechanism for both the neurological (such as encephalitis and encephalopathy; Costello and Dalakas, 2020; Ellul et al., 2020) and psychiatric (such as psychosis and delirium; Hosseini et al., 2020; Rogers et al., 2020; Steardo et al., 2020b) manifestations in COVID-19. Autoimmune responses can be induced by viral pathogens, possibly through “molecular mimicry” (Cappello et al., 2020; Lucchese and Floel, 2020) and breakdown of physiological barriers (Alam et al., 2020; Buzhdygan et al., 2020), in which unleashed autoimmune cells cross-react to exposed autoantigens, hence causing autoimmune pathology. As an immune privileged site, nervous tissue is most vulnerable for autoimmune attack, which instigates various neurological and psychiatric diseases, such as the demyelinating multiple sclerosis and Guillain-Barré syndrome (Giovannoni and Hartung, 1996), as well as the recently depicted autoimmune encephalitis (Dalmau et al., 2007; Granerod et al., 2010; Crisp et al., 2016) and autoimmune psychosis (Kayser et al., 2013; Al-Diwani et al., 2019; Pollak et al., 2020). Production of antiphospholipid autoantibodies was observed in some COVID-19 patients (Zhang et al., 2020); these autoantibodies can cause coagulopathy and cerebral infarction, conditions that have been frequently reported in victims of severe COVID-19 (Chen et al., 2020; Huang et al., 2020; Mao et al., 2020; Zhang et al., 2020) (Figure 1). Furthermore, several cases of Guillain-Barré syndrome (Arnaud et al., 2020; Padroni et al., 2020; Sedaghat and Karimi, 2020; Tiet and AlShaikh, 2020; Zhao et al., 2020) and Miller-Fisher syndrome (Gutierrez-Ortiz et al., 2020) associated with COVID-19 have been reported across the world.

As key components in compartmentalised immune responses of the CNS, both astrocytes and microglia contribute to CNS autoimmunity by regulating autoantigen-presentation, BBB or blood-cerebrospinal fluid (CSF) barrier breakdown and autoantibody leakage, as well as adaptive T- or B- cell activation for autoantibody production (Figure 1) (Ikeshima-Kataoka, 2016; Baecher-Allan et al., 2018; Dong and Yong, 2019). At this stage, it is necessary to understand whether COVID-19 patients who have recovered from cerebral infarction or sepsis might be more prone to producing autoantibodies and hence vulnerable to developing autoimmune encephalitis or psychosis in their later lives. Longitudinal clinical research would provide valuable knowledge in this sense. Besides, potential glia-mediated molecular mechanisms underlying the COVID-19 autoimmunity should also be considered in future animal research, such as disruption of the BBB and hence exposure of brain antigens to the peripheral immune system, enhanced CNS antigen-drainage via meningeal lymphatic and/or glymphatic systems, and intracranial antigen-presentation by astrocytes and microglia to infiltrated adaptive immune cells (Engelhardt et al., 2017). Currently, although the involvement of neurotropism and autoimmunity was suggested for SARS-CoV-2 and COVID-19 neuropathies (Costello and Dalakas, 2020; Hosseini et al., 2020), there has been no solid clinical or preclinical evidence of autoantibodies positively detected in the CSF or blood of COVID-19 sufferers to support this notion.

It should also be cautioned that although autoimmune response was observed in COVID-19, whether this is a transient event or even an epiphenomenon remains unclear since the underlying pathological mechanisms have not been characterised yet. Patients already affected by autoimmune diseases are, arguably, more susceptible for developing cytokine storm that can initiate or exacerbate autoimmune neurology (Askanase et al., 2020). Besides, cancer patients may also be a high-risk subgroup for both genetic and therapeutic reasons, considering that the most common autoimmune encephalitis originally discovered as a paraneoplastic syndrome occurs in 1–10% of malignant cancer patients, who usually receive immune-suppressive therapies such as corticosteroids (Dalmau et al., 2007). Among patients with autoimmune encephalitis, some present psychosis as their first or sometimes even the sole symptom, especially in the case of anti-N-methyl-D-aspartate (NMDA) receptor encephalitis (Kayser et al., 2013; Al-Diwani et al., 2019; Pollak et al., 2020). On the other hand, autoantibodies are not all pathogenic, can even circulate in healthy subjects (Ehrenreich, 2018), and therefore increased autoimmunity may not be specific for COVID-19.

In summary, whether SARS-CoV-2-derived viral protein can induce autoantibodies and whether this underlies the mechanisms of the observed demyelination or psychosis in COVID-19 warrant future research toward this direction using samples from COVID-19 patients or related animal models.

## Maternal-Foetal Transmission and COVID-19

Relations between COVID-19 and pregnancy are generally unknown because only a few cases have been hitherto documented, showing foetal distress and preterm delivery (Di Mascio et al., 2020; Matar et al., 2020). Preterm birth <37 weeks and caesarean delivery is common in pregnant women affected with COVID-19 (Di Mascio et al., 2020; Matar et al., 2020; Mullins et al., 2020). Premature labour, preeclampsia and foetal growth restriction were also observed (Di Mascio et al., 2020). Perinatal death and admission of newborns to neonatal intensive care unit were additionally higher. Infection with SARS-CoV-2 during the first trimester, evoked, in some cases, spontaneous abortion. *In utero* transmission has been described (Vivanti et al., 2020); in this instance the SARS-CoV-2 was detected in the amniotic fluid collected prior to the rupture of membranes. The infected neonate presented neurological manifestations soon after birth, which included a bilateral gliosis suggesting an early-life impact of COVID-19 on microglial (and astrocytic) status. Priming of microglia early in life may imprint the descendant's brains and thus cause a secondary event to increase their physiopathological reactions toward a subsequent stimulus. This over reaction may not be as beneficial as immunological memory, instead altering the beneficial physiological and immune functions of microglia on a long-term. It has recently been shown that oligodendrocytes and oligodendrocyte precursor cells, surviving an acute coronavirus (mouse hepatitis virus) infection, persist in the CNS for at least 150 days (Pan et al., 2020). These surviving cells express major histocompatibility complex (MHC) class I and other genes associated with a CNS inflammatory response and an extended inflammatory cell infiltration. Besides a direct transplacental transmission, SARS-CoV-2 maternal infection may have long-term consequences for the brain immunity of the descendants and thus may exert neurodevelopmental, neurological or neuropsychiatric consequences that are yet to be determined.

The risk of developing neurodevelopmental, neurological or neuropsychiatric disorders in offspring of COVID-19-infected pregnant women is notable. Maternal infection alters neurodevelopment thus contributing to the aetiology of disorders such as autism spectrum disorder, cerebral palsy, juvenile epilepsy and schizophrenia that emerge during childhood or adolescence (Knuesel et al., 2014; Zeidan-Chulia et al., 2014; Estes and McAllister, 2016). MIA and its consequences on the CNS have been documented for diverse pathogens such as influenza or bacterial infections, implicating fevers, cytokine storms, and abnormal immune responses in triggering or sustaining a systemic inflammatory state (Reisinger et al., 2015; Estes and McAllister, 2016). However, several questions remain unaddressed regarding the impact of MIA on neuroinflammation and microglia especially during brain development in the offspring (Madore et al., 2016). The MIA-generalised inflammatory state is expected to modulate microglia, including their phenotypes and functions that are essential during development to correctly shape and maintain the synaptically-connected neuronal networks. As previously characterised in other maternal infection models, MIA-exposed microglia increase their expression of MHC class II that is coupled with an altered microglial transcriptome and with a reduced phagocytic and chemotactic activity (Mattei et al., 2017; Antonson et al., 2019; Purves-Tyson et al., 2019; Thion et al., 2019). Thus, an infection by SARS-CoV-2 during pregnancy may lead to deleterious effects on microglial maturation, phenotypes and functions during neurodevelopment, and consequently increase the offspring vulnerability to developing later-on CNS disorders, the particular ones depending on when the infection occurs during gestation (Knuesel et al., 2014). These findings emphasise the importance of sizing the vulnerability and studying the potential neurodevelopmental impact of COVID-19 on the new generation. If SARS-CoV-2 leads to major increase of debilitating disorders in exposed offspring it may have profound socio-economic burden for years from now.

Perinatal environment of SARS-CoV-2-infection impacting on neurodevelopmental pathologies is also preponderant. With the increase of preterm births and admissions to the neonatal intensive care unit, COVID-19 may add to the perinatal stress that is known to be deleterious on brain development (Schepanski et al., 2018; McPherson et al., 2020). Indeed, many epidemiological studies suggest that early-life adversity can be associated with an increased occurrence of neurodevelopmental, neurological or neuropsychiatric disorders later in life (Halligan et al., 2007; Lupien et al., 2009; Knuesel et al., 2014). Moreover, the detrimental consequences of early-life stress exposure on microglial maturation are multiple and thus would alter profoundly microglial physiological activities during development (Paolicelli and Ferretti, 2017). Another factor to consider is an increase in rate of caesarean deliveries. Most of the *in utero* exposed to COVID-19 were delivered by neonates caesarean section (Di Mascio et al., 2020; Matar et al., 2020), while it has been shown that caesarean section vs. vaginal deliveries may seriously impact infant immune maturation with consequences on the brain development (Curran et al., 2015a,b; Deoni et al., 2019). For instance, caesarean section-born babies were found to lack strains of commensal bacteria required to modulate immunity (Shao et al., 2019). There is a bidirectional crosstalk between the gut and the brain, notably exerted through peripheral immune cells and the vagus nerve, which may influence disease pathogenesis by acting on microglial maturation, function and reactivity to challenges (Erny et al., 2015; Thion et al., 2018; Cryan et al., 2019). It would thus be relevant to determine whether and how COVID-19 might impact on the regulation of microglial maturation and functions via a non-optimal gut microbiome composition as a result of the caesarean section delivery. Further prospective and longitudinal studies in children born from SARS-CoV-2-infected mothers are urgently needed to answer these pressing questions.

SARS-CoV-2 maternal infection in combination with a non-adequate perinatal environment may have long-term consequences for the offspring's brain immunity. SARS-CoV-2 maternal infection may lead to an increased risk of developing neurodevelopmental, neurological or neuropsychiatric disorders later-on in life, making it critical to closely follow up and eventually prevent the onset of adverse consequences for human brain health in the descendants.

## Aged Neuroglia Increases the Vulnerability of the Brain to COVID-19

Ageing increases the vulnerability to SARS-CoV-2 and severity of COVID-19; the death rate in old population attained ~10% compare to overall death rate of 0.66% (Mahase, 2020). Similarly, neurological and cognitive deficits seem to be on increase in elderly with COVID-19 history (Devita et al., 2020; Varatharaj et al., 2020). Brain as an organ sustains ageing remarkably well; cognitive excellence sustains into ages when physical abilities are remarkably affected (Verkhratsky, 2019). However, the cognitive longevity is severely affected by neurodegenerative diseases, most of which do not have obvious genetic links but are age-dependent: ageing is the major risk factor for such disorders. There are increasing arguments for glial cells being responsible for defining the cognitive longevity and cognitive reserve (Rodriguez-Arellano et al., 2016).

Ageing is associated with accumulation of dystrophic forms of glial cells, with loss of homeostatic and defensive support (Figure 2), with decrease in neurogenesis (which is the function of radial stem astrocytes, also influenced by neuroinflammation and microglial phagocytosis) and with failure of oligodendroglial precursors to sustain myelination (Streit et al., 2004; Sierra et al., 2014; Verkhratsky et al., 2015a, 2019; Tay et al., 2017b; Vanzulli et al., 2020). Glial cells in the older brains reduce their ability to mount gliotic response hence facilitating brain damage by imported pathogens (Verkhratsky et al., 2015b). Furthermore, the astroglia-based glymphatic waste collection system (Iliff et al., 2012) declines with age, which affects clearance of numerous by-products and toxic substances (Kress et al., 2014). All these widespread glial abnormalities, although being barely detectable in normal life, make the brain much more vulnerable to systemic pathologies and infectious damages. There is a well-defined link between systemic inflammation or major systemic diseases and cognitive deficits: systemic pathologies promote neurodegenerative process and accelerate cognitive decline (Perry et al., 2003). Thus, COVID-19 is not only deadly for old people, it may also have grave cognitive consequences.

## Conclusion: Glial Performance Might Define the Neurological Outcome of COVID-19

Overall, the findings discussed in this review indicate that SARS-CoV-2 has a strong neurotropism that can result in both immune deficiencies, due to the burn-out of innate and adaptive immunities, and autoimmunity, due to the loss of self-tolerance by the immune system. These changes in the brain are mediated by astrocytes and microglia, which are the first responders of the brain to trauma, injury, infection and disease. The exposure to viral infection causes astrogliosis, a reprogramming of these cells associated with neuroprotection that also disrupts the BBB, inducing failure of this very important protection barrier against the deleterious consequences of systemic inflammation including the entry of peripheral immune cells and DAMPs into the brain. These effects are particularly pronounced in vulnerable areas of the brain lacking a BBB such as the CVOs. Similarly, infected microglia become primed, a phenotypic shift in which they display an exaggerated release of pro-inflammatory mediators and an aberrant phagocytic activity that induces neurodegeneration (killing neurones through their live engulfment named “phagoptosis”; Brown and Neher, 2014), synaptic loss (by several modalities that include “trogocytosis,” i.e., nibbling of small pieces of axon terminals; Weinhard et al., 2018), and demyelination (Traiffort et al., 2020), reflecting a loss of their beneficial functions in neuroprotection, synaptic maintenance, activity and/or plasticity, as well as myelination.

These over-reactions of glial cells exposed to viral infection, notably with SARS-CoV-2, are further exacerbated when microglia are primed or immunologically alerted by the previous challenges they have encountered, which especially occurs in the areas of the brain devoid of a BBB such as the CVOs, whether these challenges result from comorbidity, chronic stress, dietary imbalance and gut dysbiosis, sleep disturbances, pollution, and/or other known epidemiological risk factors. Over time, the glial alterations result in cellular stress, ultrastructural alterations to organelles (e.g., mitochondria), and functional impairments that become progressively more pronounced during adulthood into ageing. With ageing, astrocytes and microglia become altered in various ways, adopting reactive and primed, but also dystrophic, senescent, dark, and disease-associated phenotypes that fail to maintain proper BBB function, brain homeostasis, neuronal circuit maintenance, activity and plasticity, sensorimotor gating, cognitive flexibility, judgment, decision making, and other behavioural outputs, thus triggering and accelerating the onset of cognitive decline, dementia, social dysfunction, depression, psychosis, other neuropsychiatric and neurodegenerative conditions with age, leading to premature death, across all walks of life. On the other end of the spectrum, offspring exposed to MIA induced by SARS-CoV-2 infection might be at particularly high risk of developing various neurodevelopmental, neurological and psychiatric conditions as a consequence of COVID-19 exposure, due to systemic inflammation (at a time when the BBB is still forming), gut-brain axis dysfunction and neuroinflammation leading to compromised physiological and immune glial cell functions throughout life.

## Author Contributions

All authors listed have made a substantial, direct and intellectual contribution to the work, and approved it for publication.

## Conflict of Interest

The authors declare that the research was conducted in the absence of any commercial or financial relationships that could be construed as a potential conflict of interest.
